# Synergistic compatibilization of PA12/PPO blends by PPO-*g*-MA and graphene oxide: mechanical, thermal, and flame-retardant properties

**DOI:** 10.1039/d5ra06435j

**Published:** 2025-11-06

**Authors:** Pham The Long, Nguyen Thi Ngoan, Vu Thi Hoang Anh, Nguyen Huu Dat, Nguyen Vu Giang, Tran Thi Y Nhi, Luong Nhu Hai

**Affiliations:** a Center for High Technology Research and Development, Vietnam Academy of Science and Technology 18 Hoang Quoc Viet, Nghia Do Hanoi 10000 Vietnam luonghai76@gmail.com long.pth90@gmail.com; b Institute of Materials Science, Vietnam Academy of Science and Technology 18 Hoang Quoc Viet, Nghia Do Hanoi 10000 Vietnam; c Graduate University of Science and Technology, Vietnam Academy of Science and Technology 18 Hoang Quoc Viet Street, Nghia Do Hanoi 10000 Vietnam; d Institute of Chemistry, Vietnam Academy of Science and Technology 18 Hoang Quoc Viet, Nghia Do Hanoi 10000 Vietnam

## Abstract

In this study, polyamide 12 (PA12)/poly(2,6-dimethyl-1,4-phenylene oxide) (PPO) blends with a mass ratio of 90/10 were compatibilized using maleic anhydride-*grafted*-PPO (PPO-*g*-MA), graphene oxide (GO), and their combinations to enhance the interface compatibility and improve the mechanical, thermal, and flame-retardant properties of the PA12/PPO blends. The effects of the compatibilizers on the blends' morphology, mechanical behavior, dynamic mechanical properties, thermal stability, and flame retardancy were investigated. The glass transition temperatures of the blends using the compatibilizers showed shifts compared to the neat blend, indicating that the compatibility of PA12 and PPO in these blends was improved. The dual compatibilizer system significantly enhanced the dispersion and interfacial interaction, leading to synergistic improvements, thereby improving the mechanical strength, thermal stability, and flame retardancy of the blend compared to the blend using each compatibilizer individually. The blend sample using 3% PPO-*g*-MA and 1% GO achieved a tensile strength of 48.6 MPa, impact strength of 47.3 kJ m^−2^ compared to 29.3 MPa and 33.1 kJ m^−2^ of the neat PA12/PPO blend, respectively. The thermal stability of the blends was also enhanced by the simultaneous addition of both PPO-*g*-MA and GO. Besides, the enhanced thermal stability was directly reflected in flame resistance, with the limiting oxygen index (LOI) increasing from 21.9% to 24.6% and the UL-94 rating improving from V2 to V1.

## Abstract 2

Schematic description of the synergistic compatibility mechanism of GO and PPO-*g*-MA in PA12/PPO blends.

## Graphical abstract

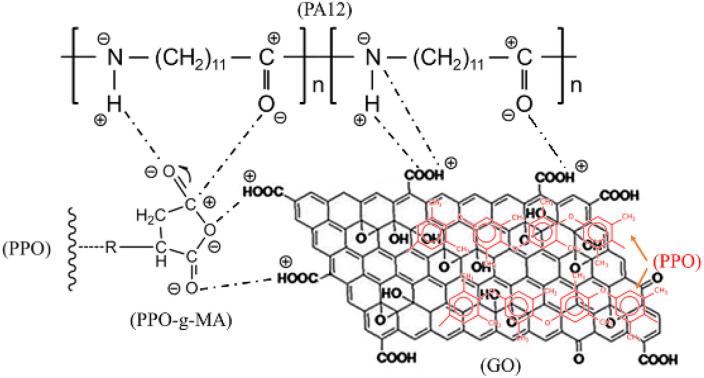

## 1 Introduction

Polyamide 12 (PA12) and poly(2,6-dimethyl-1,4-phenylene oxide) (PPO) are two engineering thermoplastics that are widely used in many industrial fields due to their excellent mechanical properties, electrical properties, and chemical resistance. PA12 is also known for its high toughness, abrasion resistance, and ease of processing, flexibility, and especially the lowest water absorption compared to other polyamides,^1–3^ making it suitable for automotive, electrical 3D-printing applications, *etc.*^1–8^ PPO has excellent inherent dimensional stability, thermal stability, and flame retardancy, making it a valuable polymer for high performance applications.^9–13^ The main disadvantages of PA12 are its relatively low thermal stability, poor dimensional stability, and poor flame retardancy, while with PPO, it is the high glass transition temperature (*T*_g_) and low processability that make PPO difficult to process. Blending PA12 with PPO offers a promising solution to combine their complementary properties, potentially producing materials with improved mechanical strength, thermal stability, and flame retardancy. However, differences in structure and processing temperatures make them difficult to blend, and thermodynamic incompatibility leads to phase separation and weak interfacial bonding, which reduces the mechanical and thermal performance of the blend. Therefore, enhancing the compatibility between PA12 and PPO is crucial to exploiting the full potential of these blends.

A common approach to improving the compatibility and miscibility of polymer blends is to add a compatibilizer, which can be a copolymer or a modified polymer.^14–17^ In general, the added compatibilizers are compatible with both phases, thereby preferentially separating at the interface and ensuring strong interfacial adhesion. Maleic anhydride-*grafted*-PPO (PPO-*g*-MA) is a widely studied compatibilizer that adds reactive anhydride groups capable of forming covalent or hydrogen bonds with the amine or carboxyl groups in the PA12 chain, thereby improving interfacial adhesion and reducing phase separation. The PPO backbone in PPO-*g*-MA ensures chemical affinity with the PPO phase, facilitating better dispersion and morphological stability of these blends.^15–17^ However, this compatibilization method has some disadvantages: copolymers themselves are of little benefit to the strength and heat resistance of polymer blends because they are organic polymers. In some cases, the addition of copolymers even weakens some properties of the blend.^18–21^ Modified polymer compatibilizers often have molecular chain breaks during the fabrication process, resulting in their molecular weights not being as high as the original polymers, thus weakening the performance of these polymers.

In addition to compatibilizers, graphene oxide (GO) also has the potential to improve the compatibilization of polymer blends with different polarities, such as PA12 and PPO, due to its unique two-dimensional structure, high aspect ratio, and excellent mechanical and thermal properties. Many studies have shown that GO consists of hydrophobic π domains on the basal plane and contains many hydrophilic functional groups (hydroxyl, carboxyl, *etc.*) at the edges.^22–24^ Therefore, GO exhibits amphiphilic properties, can form strong hydrogen bonds with the amide and amine groups of PA chains,^25^ and can adsorb nonpolar polymers on their basal planes through π–π stackings or hydrophobic interactions.^26^ Furthermore, PPO has a structure consisting of many aromatic rings, so it is very easy to be compatible with GO sheets. Therefore, this nanofiller has the potential to be used as a compatibilizer for PA/PPO blends. The addition of GOS to polymer blends not only improves their compatibilization but also enhances their mechanical and thermal performance. The main problem with this method is the poor dispersion of GO in the polymer matrix. GO sheets tend to agglomerate within the matrix.^24,27–29^ This aggregation can drastically reduce the properties of the polymer. Besides, the low usable nanofiller content can limit the effect of GO in improving the compatibility of the blend.

Based on the analysis of the characteristics of each type of compatibilizer, it can be seen that each method has its own advantages and disadvantages, and the combination of organic polymer compatibilizers with inorganic compatibilizers, such as GO can be a potential method to improve the compatibilization of polymer blends. Although many studies have been conducted on individual compatibilizers or nanofillers, the combined effect of these two methods as dual compatibilizers in PA12/PPO blends has not been fully explored. This study aims to investigate the synergistic effects of PPO-*g*-MA and graphene oxide on the mechanical properties, thermal stability, flame retardancy, and morphological characteristics of PA12/PPO blends. These results are expected to contribute to the improvement of the performance of PA12/PPO blends with improved compatibilization and meet the requirements for advanced industrial applications.

## 2 Experimental

### 2.1. Materials

Polyamide 12 (PA12) with a trade name of UBESTA 3030-JFX1 was obtained from UBE Corporation (Japan) – density: 1.03 g cm^−3^ and flow index (235 °C; 2.16 kg): 7.4 g/10 min. Polyphenylene oxide (PPO) was purchased from SABIC, Saudi Arabia has a density of 1.13 g cm^−3^. Polyphenylene ether-*grafted*-maleic anhydride (PPO-*g*-MA) with a density of 1.06 g cm^−3^ and a grafting ratio of 0.5÷1 wt% was supplied by Fine-Blends Company, China. Graphene oxide (GO) with an average diameter of 7.5 μm was supplied by Changzhou Sixth Element Materials and Technology Ltd, P.R. China. Other solvents were purchased from commercial suppliers and used as received.

### 2.2. Preparation of PA12/PPO blends

Solvent blending of PPO and GO to prepare PPO/GO master batch. GO was dispersed in THF with the aid of sonication. Afterward, PPO was added to the suspension. After agitation at 50 °C for 2 h and sonication at 45 °C for another 1 h, the mixture was coagulated with methanol. The flocculent was filtered under vacuum, and then vacuum-dried at 40 °C for 12 h, yielding a PPO/GO masterbatch. Masterbatch or neat PPO and PPO-*g*-MA were added into PA12 and were melt-blended using a HAAKE internal mixer operating at 235 °C, mixing speed of 50 rpm for 5 min to obtain a series of PA12/PPO blends. Then the material mixture in the molten state was hot pressed using a hydraulic press heated at 250 °C to form flat sheets with a thickness of 1.5–2 mm. Samples were cooled to room temperature and allowed to stabilize slightly for at least 24 h before material properties were examined. The abbreviations and component loadings of the blends preparation are listed in Table 1. In this work, the PA12/PPO blend weight ratio was kept at 90/10 (ref. 30) with the compatibilizers being PPO-*g*-MA and GO.

**Table 1:** Sample abbreviations and component loadings of PA12/PPO blend

Sample	PPO-*g*-MA content (wt%)	GO content (wt%)
BP	0	0
BPC	3.0	0
BP/GO-0.5	0	0.5
BPC/GO-0.5	3.0	0.5
BPC/GO-1.0	3.0	1.0

### 2.3. Characterization

Measurements of tensile strength were performed at a tensile speed of 10 mm min^−1^ on a Zwick Tensiler 2.5 (Germany) device according to ASTM D 638 standards; data are averaged across 5 measurement samples. The notched Izod impact strength characteristics were measured using an impact testing machine, Testresources (USA), according to ASTM D256 standard at room temperature. The morphology of the surface structure of the blend material was determined on a field emission scanning electron microscope (FE-SEM) S-4800 (Hitachi, Japan). Evaluate the thermal properties of materials on a thermogravimetric analyzer TGA 209F, (Netzsch, Germany) with a heating rate of 10 °C min^−1^, under nitrogen atmosphere, from room temperature to 700 °C. Differential scanning calorimetry measurement was performed on a DSC 204F1 equipment (PerkinElmer, USA) with a heating rate of 10 °C min^−1^ and temperature ranging from 0 to 300 °C. Dynamic mechanical analysis (DMA) was performed on a DMA-1 system (Mettler Toledo, Switzerland) in a torsion mode at a frequency of 1 Hz and a strain of 0.1%. Each sample was cut into a 50 × 10 × 2 (mm) rectangle. The scanning temperature was varied from 40 to 170 °C at a heating rate of 4 °C min^−1^. The evolution of the damping factor (tan *δ*) as a function of temperature was recorded. The flammability rating of the samples was determined using the UL-94 test procedure. The limiting oxygen index (LOI) was determined according to the test procedure of ASTM D2863.

## 3 Result and discussion

### 3.1. Morphology of PA12/PPO blends

**Fig. 1:**
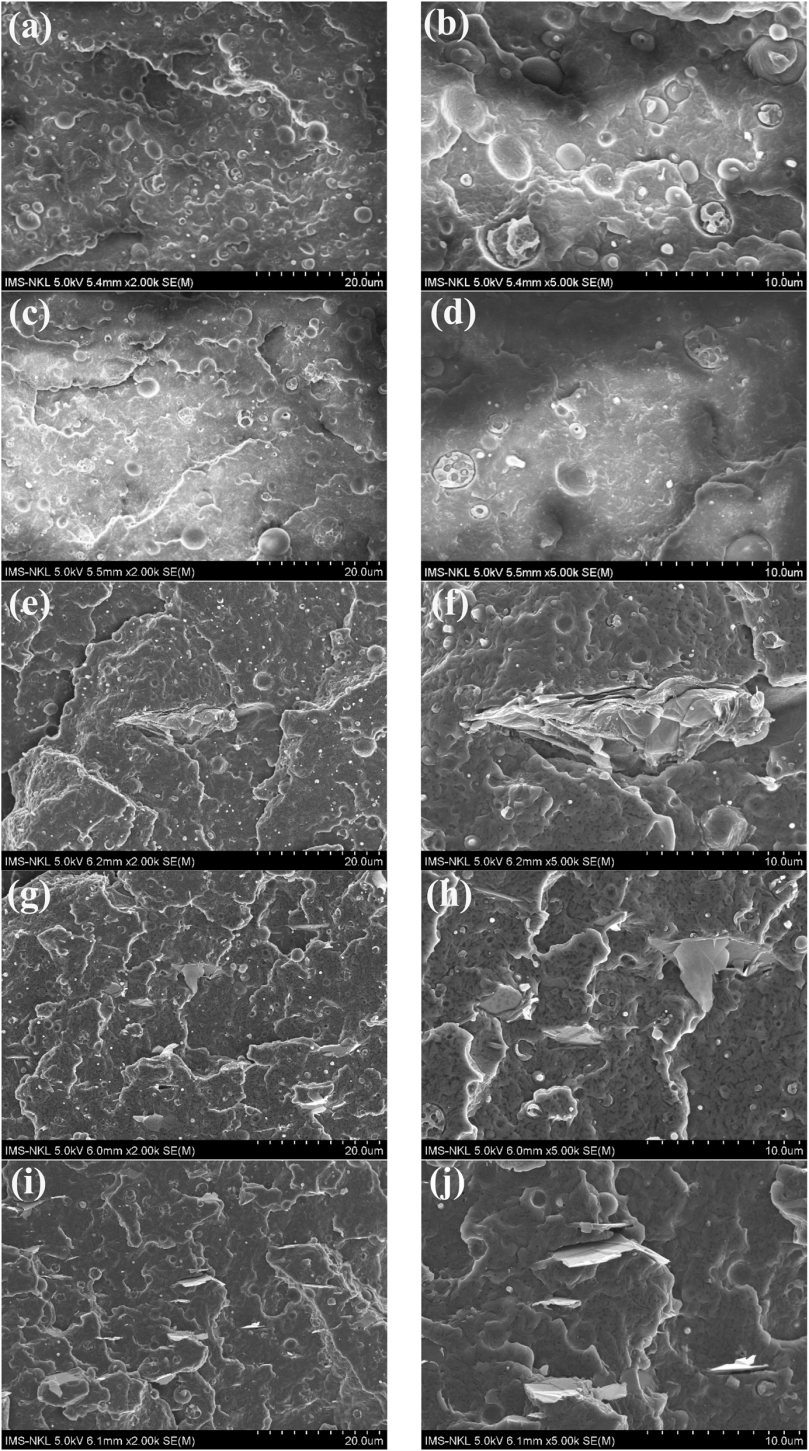
SEM image fracture surface of PA12/PPO blend samples: BP (a and b), BPC (c and d), BP/GO-0.5 (e and f), BPC/GO-0.5 (g and h) and BPC/GO-1.0 (i and j).

Fig. 1 presents the SEM micrographs of the cryogenically fractured surfaces of PA12/PPO blends with different compatibilizer systems. In the incompatible BP sample (PA12/PPO = 90/10), a distinct phase-separated morphology was observed. The PPO phase appears as dispersed spherical domains with large diameters, non-uniform distribution, and smooth boundaries, indicating poor interfacial adhesion between the PA12 matrix and the PPO phase.

**Fig. 2:**
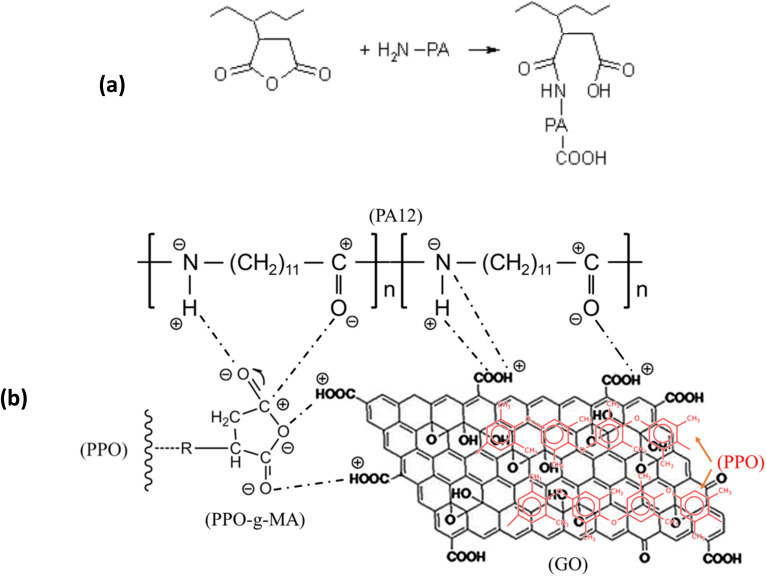
Interfacial interaction between PA12 and anhydride group in PPO-*g*-MA (a) and schematic description of the synergistic compatibility mechanism of GO and PPO-*g*-MA in PA12/PPO blends (b).

When 3 wt% PPO-*g*-MA was introduced into the blend (sample BPC), the PPO domain size was significantly reduced, and its distribution became more homogeneous. The interfacial region also became less distinct, suggesting improved interaction between PA12 and PPO. This result demonstrates that PPO-*g*-MA, bearing maleic anhydride groups, can react with the amine end groups of PA12 chains to form *in situ* copolymers at the interface (description in Fig. 2a).^31,32^ Such *in situ* reactions improve phase adhesion and help to refine the morphology, similar to the mechanism reported.

The addition of 0.5 wt% GO to the incompatible blend (BP/GO-0.5 sample) also reduced the PPO domain size and improved the dispersion. Compared to the BP sample, the morphology of BP/GO-0.5 showed slightly smaller PPO domains and fewer interfacial voids. This effect may be due to the amphiphilic nature of graphene oxide sheets, which tend to concentrate at the interface between insoluble phases and act as a physical compatibilizer. GO also acts as a barrier, limiting domain growth during melt mixing. However, it is also possible to observe GO agglomerates in the blend matrix. This is an inevitable drawback of nanofillers and limits the compatibilization efficiency of this type of additive.

The most uniform and refined morphology was observed in the samples containing both PPO-*g*-MA and GO (BPC/GO-0.5 and BPC/GO-1.0). In these samples, the PPO phase was highly dispersed in the PA12 matrix with very small and uniformly distributed domains. The interfacial boundaries were smooth and continuous, indicating strong adhesion between the two phases. The synergistic effect between chemical compatibilization by PPO-*g*-MA and physical compatibilization by GO was clearly demonstrated in these samples.

Fig. 2 illustrates the compatibilization mechanism of PPO-*g*-MA and GO in PA12/PPO blends. PPO-*g*-MA interacts with PA12 through the *in situ* reaction between the maleic anhydride groups on PPO-*g*-MA and the terminal amine groups of PA12 (Fig. 2a). In addition, hydrogen bonding can occur between the amide groups of PA12 and the anhydride groups of PPO-*g*-MA, while the PPO backbone of the compatibilizer remains miscible with the PPO phase, thereby effectively bridging the two polymers at the interface. Meanwhile, graphene oxide (GO) promotes additional interfacial adhesion through multiple interactions. The oxygen-containing functional groups on GO form strong hydrogen bonds with both the amide and amine groups of PA12, while its aromatic surface interacts favorably with the aromatic backbone of PPO through π–π stacking and hydrophobic interactions (Fig. 2b). PPO-*g*-MA also interacted effectively with GO and improved the diffusion of GO from the masterbatch with PPO into the PA12 matrix, thereby limiting the agglomeration of GO in the blend. The combination of both agents enhanced dispersion, prevented coalescence, and stabilized the blend morphology during melt mixing.

### 3.2. Mechanical properties of PA12/PPO blends

**Fig. 3:**
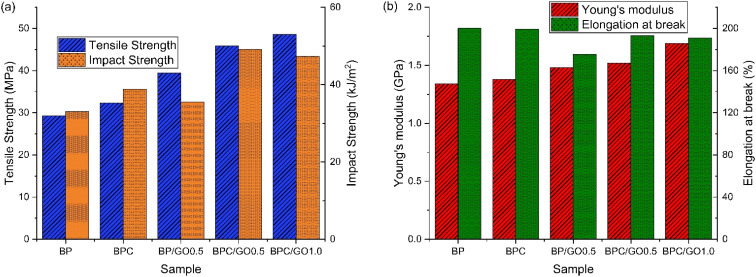
Tensile strength, impact strength (a) and Young's modulus, elongation at break (b) of PA12/PPO blends with different compatibilizer system.

The tensile strength results of the PA12/PPO blends are presented in Fig. 3. The incompatible BP sample exhibited the lowest tensile strength, reaching only 29.3 MPa, with a Young's modulus of 1.34 GPa and an elongation at break of 200.1%. This low value reflects the strong phase separation between PPO and PA12, which leads to poor interfacial adhesion and inefficient stress transfer across the interface. As confirmed in the SEM images, the presence of large PPO domains with smooth, distinct boundaries contributes to early crack initiation under tensile stress.

With BPC sample, the tensile strength improved significantly to 36.3 MPa, while the modulus slightly increased to 1.38 GPa and the elongation at break remained almost unchanged (199.3%). This improvement was due to the *in situ* formation of chemical bonds between the maleic anhydride groups on PPO-*g*-MA and the terminal amine groups of PA12 combined with the formation of hydrogen bonds between the amide groups and the anhydride groups on the compatibilizer. These covalent and hydrogen bonds enhance interfacial adhesion, enabling better stress transfer from the matrix to the dispersed phase. However, PPO-*g*-MA does not have high mechanical strength as a consequence of the fabrication process, which somewhat reduces the mechanical strength of the blend. Therefore, the tensile strength of the BPC sample was not significantly improved compared to the original blend sample.

For the blend sample reinforced with 0.5 wt% graphene oxide (BP/GO-0.5 sample), the tensile strength increased significantly, reaching 39.4 MPa, accompanied by a higher modulus of 1.48 GPa but a noticeable reduction in elongation to 175.5%. This is due to the amphiphilic nature of the GO sheets, which acts as a physical compatibilizer and interact well with both PA12 and PPO. Furthermore, GO acts as a reinforcing nanofiller with high stiffness and large specific surface area, which helps to limit the mobility of the polymer chains and enhance the overall strength of the material. However, the restricted chain mobility and partial agglomeration of GO observed in FE-SEM image suggests that the effect of GO on the PA12/PPO blend may be partially impaired, reflecting the typical conflicting effect between stiffness and toughness when introducing nanofillers.

Most notably, when PPO-*g*-MA and GO were combined (BPC/GO-0.5 and BPC/GO-1.0), the tensile strength increased significantly, reaching a maximum value of 48.6 MPa for the sample containing 1.0 wt% GO, and the modulus further increased to 1.69 GPa, while the elongation at break slightly decreased to 191.1%. This shows a strong synergistic effect between chemical compatibility (by PPO-*g*-MA) and physical reinforcement (by GO). At the same time, the specific interaction between PPO-*g*-MA and GO has the effect of improving the disadvantages of these two compatibilizers: PPO-*g*-MA supports better dispersion of GO in the resin matrix, limiting agglomeration (observed in Fig. 1g, h and i, j), conversely, GO can diffuse into the PPO-*g*-MA matrix, acting as a reinforcement for this modified polymer, thereby improving the overall mechanical properties of the blend. Improved interface bonding, more refined morphology, and more efficient load transfer, combined with the improved reinforcing effect of the nanofiller, contribute to significantly enhanced tensile strength. These results also demonstrate that the dual-compatible system provides an optimal balance between stiffness and toughness, achieving reinforcement and improved strength while ensuring that the material's flexibility is not significantly affected.

The impact strength of the blends showed a similar trend to that of tensile strength. The incompatible BP sample exhibited the lowest impact resistance, with a value of 33.1 kJ m^−2^. This is mainly due to the poor compatibility between PA12 and PPO, which results in distinct phase separation and weak interfacial adhesion. Under impact loading, these interfacial defects serve as stress concentrators, facilitating crack initiation and propagation.

Unlike the tensile strength, the inclusion of PPO-*g*-MA (BPC sample) significantly increased the impact strength to 38.8 kJ m^−2^. This improvement was due to the formation of chemical bonds at the phase interface, which reduced the separation of interface bonds under dynamic loading. The reduced matrix defects effectively prevented the formation and propagation of cracks, thereby enhancing the impact resistance.

On the other hand, the use of 0.5 wt% GO (BP/GO-0.5 sample) resulted in an impact strength of 35.5 kJ m^−2^, a slight increase compared to the initial blend. Although GO contributes to the improvement of the structural morphology, the enhancement of the compatibility between PA12 and PPO, and the reduction of defects in the resin matrix, the improvement in modulus and the reduction in elongation at break indicates that the introduction of rigid GO sheets restricted the segmental motion of polymer chains and increased material stiffness, thereby limiting its ability to dissipate energy under sudden loading, especially when not supported by additional compatibilization. Therefore, the impact strength in this case did not change significantly. In addition, the agglomeration of GO in the matrix also contributed to this result.

Similar to the trend observed in tensile strength, the combination of PPO-*g*-MA and GO resulted in a significant improvement in impact strength. This improvement may be due to the synergistic effect between the two compatibilizers. PPO-*g*-MA enhances the interface bonding and prevents crack formation, while GO acts as a defect-reducing reinforcement, preventing crack propagation. Furthermore, the fine and uniform morphology observed in these samples helps to limit stress concentration and enhance the overall toughness. As the GO content increased, the impact strength of the blend tended to decrease slightly (47.3 kJ m^−2^ of 1.0 wt% GO compared with 49.1 kJ m^−2^ of 0.5 wt% GO), which may be due to the large GO content hindering the relative movement of the polymer, making the material stiffer (Young's modulus increased to 1.69 GPa compared with 1.52 GPa of 0.5 wt% GO) and reducing the impact resistance. Nevertheless, the impact strength of BPC/GO-1.0 remains considerably higher than that of the single-compatibilized blends, confirming that the dual compatibilization mechanism effectively preserves toughness even at higher stiffness levels.

### 3.3. Thermal stability of PA12/PPO blends

**Fig. 4:**
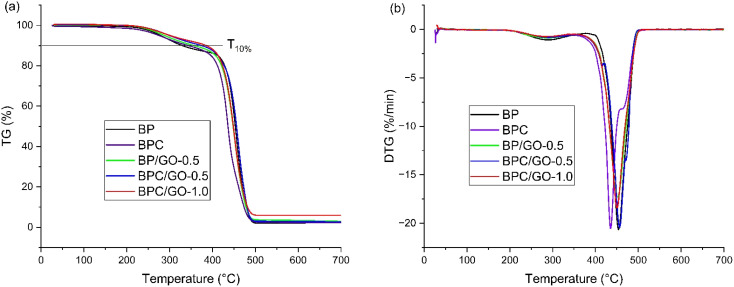
TGA curves (a) and derivative thermogravimetric curves (b) of PA12/PPO blends with different compatibilizer system.

To study the influence of different compatibilizer systems on the thermal stability of the blend material, the thermal properties of PA12/PPO blend samples were investigated by thermogravimetric analysis (TGA). The results of TG curves and derivative thermogravimetric (DTG) curves are shown in Fig. 4. In general, the decomposition of the PA12/PPO blend proceeds in two steps: depolymerization of the end-chain functional groups in the temperature range from 195 to 360 °C and cutting the main polymer chain by forming unsaturated nitriles and alkenes in the temperature range from 365 to 510 °C, the main thermal degradation step of PA12 and PPO are overlapped. The characteristic parameters of the thermal decomposition of materials are characterized by *T*_10%_, (temperature at the weight loss of 10%), maximum decomposition temperature (*T*_max_), and residual char rate at 700 °C presented in Table 2.

**Table 2:** Characteristic parameters for the thermal decomposition process of material samples

Sample	*T*_10%_ (°C)	*T*_max_ (°C)	Residual char rate (%)
BP	332.4	452.6	2.2
BPC	343.1	434.8	2.4
BP/GO-0.5	357.4	451.4	2.8
BPC/GO-0.5	371.4	453.4	2.9
BPC/GO-1.0	385.8	451.8	5.3

As shown, the immiscible BP blend exhibited the lowest *T*_10%_, at 330.6 °C, and a *T*_max_ of 452.6 °C with only 2.2% residual char. This relatively low thermal stability is due to the poor compatibility between PA12 and PPO. The phase-separated morphology facilitates the premature decomposition of the PA12 matrix and reduces the effectiveness of preventing thermal decomposition of the PPO phase. In such blends, heat transfer across the interface is inefficient, and the lack of an interfacial barrier allows volatile products to escape more easily during thermal decomposition.

The addition of 3 wt% PPO-*g*-MA (BPC sample) increased the *T*_10%_ to 343.1 °C. This is completely consistent with the morphology observation. The improved phase compatibility results in a more uniform morphology and reduced interfacial defects, thereby enhancing the thermal stability of the blend. The interfacial bonding associated with the PPO phase acts as a barrier to the thermal motion of polymer chains and delays the degradation process. However, the DTG peak shifted to a lower temperature of 434.8 °C, suggesting that decomposition of the main polymer chain occurred earlier. As a modified polymer, PPO-*g*-MA contains reactive maleic anhydride groups, shorter chain segments, and lower molecular weight compared to neat PPO resin, which are more susceptible to chain scission under heat. At this temperature range, random chain breaking and the formation of volatile fragments occur, resulting in a noticeable drop in *T*_max_ and faster mass loss. This phenomenon represents a typical drawback of functionalized polymeric compatibilizers – although they improve phase adhesion, their thermal stability is inherently inferior to that of the neat polymer.

The use of GO as a compatibilizer in the BP/GO-0.5 sample led to a more significant increase in thermal stability. The improvement is explained by the high thermal conductivity and layered structure of GO with a large contact area, which acts as an effective physical barrier that slows the diffusion of volatile degradation products and insulates the underlying material from external heat. Additionally, GO restricts the chain mobility through its strong interfacial interaction with polymer segments, leading to delayed degradation onset. The slight increase in char yield indicates the initial contribution of GO to carbonaceous residue formation during decomposition.

Remarkably, the blends containing both PPO-*g*-MA and GO (BPC/GO-0.5 and BPC/GO-1.0) showed the highest thermal stability, with *T*_10%_ values of 371.4 °C and 385.8 °C, and *T*_max_ of 453.4 °C and 451.8 °C, respectively. Although GO does not substantially alter the second-stage decomposition (as reflected by similar *T*_max_ values), it effectively mitigates the early degradation tendency caused by the lower-molecular-weight PPO-*g*-MA. These results demonstrate a clear synergistic effect between chemical and physical compatibilization mechanisms. The enhanced compatibility between PA12 and PPO and the uniform morphology facilitate the aromatic ring structure of PPO to exhibit its thermal stability advantages, while the barrier effect from GO and PPO results in a densely cross-linked and well-shielded structure with better resistance to thermal degradation. In terms of residue formation, while the samples BPC, BP/GO-0.5, and BPC/GO-0.5 exhibited only slightly increased in char yield (2.4–2.9%), the BPC/GO-1.0 sample showed a marked increase to 5.3%, indicating that a sufficiently high GO content promotes the formation of a stable carbonaceous char. Increasing GO content (0.5 to 1.0 wt%) leads to an increased *T*_10%_ value and residual char rate, which also shows that GO promotes char formation during degradation, which protects the underlying polymer from further degradation, acting as a protective layer.

This result is consistent with the SEM observation, mechanical tests above and clearly shows that the combined use of PPO-*g*-MA/GO dual compatibilizer system significantly improves the interphase interaction and miscibility of the blends, leading to the improvement of thermal stability of the blends.

### 3.4. Dynamic mechanical and differential scanning calorimetry of PA12/PPO blends

**Fig. 5:**
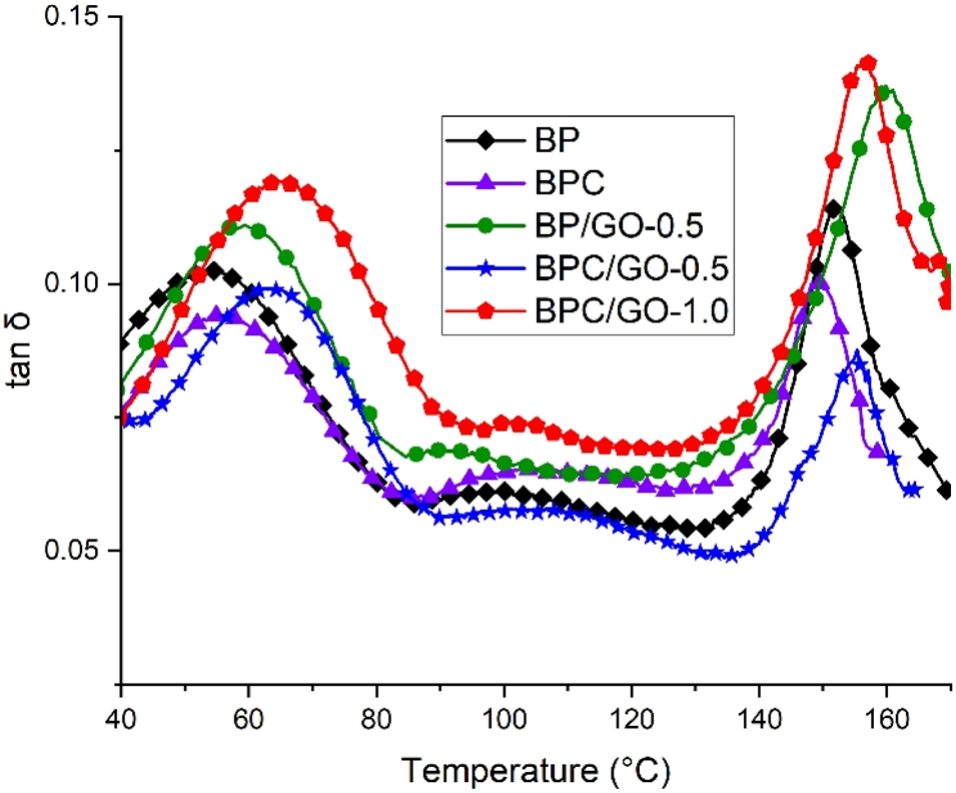
tan *δ* of PA12/PPO blends with different compatibilizer systems.

Damping factors (tan *δ*) of the PA12/PPO blends as a function of temperature are shown in Fig. 5. The incompatible blend (BP) exhibited two distinct tan *δ* peaks, corresponding to the glass transitions of PA12 and PPO phases, respectively. This is a typical behavior of immiscible polymer blends and confirms that the PA12 and PPO components remain largely phase-separated in the absence of a compatibilizer.

For the blend sample using the compatibilizer PPO-*g*-MA, both *T*_g_ values of PA12 and PPO tend to move closer together (*T*_g_ of PA12 increases, *T*_g_ of PPO decreases). This phenomenon shows that the molecular chains interpenetrating at the interface, which is considered partially miscible, indicating that the compatibility between the two phases is improved by the influence of PPO-*g*-MA. In the case of the BP/GO-0.5 sample, where GO was used exclusively as a physical compatibilizer, a different feature was observed. Both the *T*_g_ values of PA12 and PPO shifted toward higher temperatures compared to the BP sample. This may be due to the high stiffness and lamellar structure of GO, which restricts the relative motion of the macromolecular chains, thus increasing the energy required for the glass transition. In addition, the presence of GO at the interface and within the PA12 matrix hinders the fractional expansion; the enhanced compatibility between the PA12 and PPO phases, combined with the specific effect of GO, resulted in a greater increase in *T*_g_ of PA12 compared to the BPC sample. These results are consistent with the reinforcing and motion-restraining effects of GO observed in previous studies.^33,34^

Interestingly, the blends containing two compatibilizers, PPO-*g*-MA and GO (BPC/GO-0.5 and BPC/GO-1.0) showed more obvious changes in glass transition behavior. In particular, the *T*_g_ of PA12 increased significantly compared to the BPC sample, while the *T*_g_ of PPO decreased slightly further. This behavior indicates that GO not only acts as a nanofiller but also plays an important role in enhancing the miscibility between PA12 and PPO. The increased *T*_g_ of PA12 indicates that the PA12 chains are more restricted in movement due to the improved bonding and confinement by GO sheets, while the improved compatibility makes the *T*_g_ of PA12 tend to move toward the *T*_g_ of PPO. On the other hand, the decreased *T*_g_ of PPO implies better dispersion of PPO in the matrix and closer interaction with PA12 chains. The resulting blend structure was better blended, confirming that GO enhances the compatibilization more effectively when used together with PPO-*g*-MA (Table 3).

**Table 3:** Glass transition temperature values of PA12/PPO blends

Sample	*T*_g1_ (°C)	*T*_g2_ (°C)
BP	53.6	152.3
BPC	54.9	149.1
BP/GO-0.5	57.0	159.8
BPC/GO-0.5	62.5	155.4
BPC/GO-1.0	64.1	156.2

Overall, the results of the dynamic mechanical analysis showed that the combination of PPO-*g*-MA and GO had a more pronounced effect on enhancing the compatibility of PA12/PPO blends than the use of each compatibilizer individually. These findings are fully consistent with the observed improvements in morphology and mechanical performance, and further verify the synergistic role of the dual compatibilizer system in PA12/PPO blends.

**Fig. 6:**
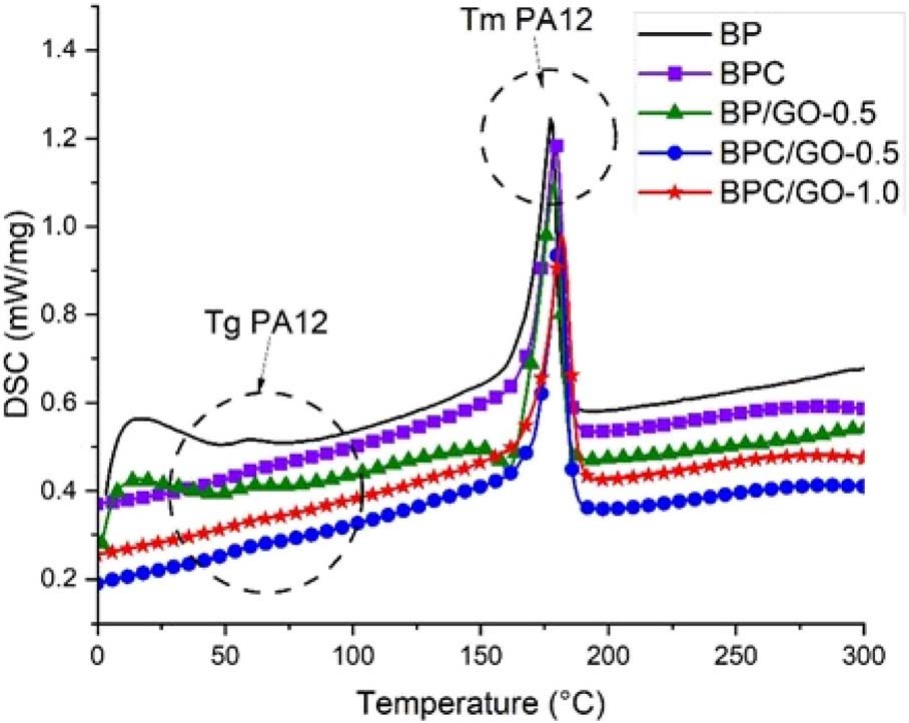
DSC curves of PA12/PPO blends with different compatibilizer system.

Fig. 6 shows DSC curves of PA12/PPO blend with temperatures from 0 °C up to 300 °C. All samples have a peak with a range from 150 to 190 °C, which represents the melting temperature of the PA12 phase. As shown in the DMA curves above, the glass transition temperature region of the PPO phase in PA12/PPO blends is about 150 to 170 °C, so this phase transition region cannot be observed on the DSC curve because it is obscured by the melting peak of PA12 phase. Therefore, only the *T*_g_ value of the PA12 phase was obtained from this measurement at the region from 55 to 65 °C. The glass transition temperature *T*_g_ and melting temperature *T*_m_ of PA12 in blends were collected and listed in Table 4.

**Table 4:** Melting temperatures (*T*_m_) and glass transition temperatures (*T*_g_) of PA12/PPO blends

Sample	*T*_g_ (°C)	*T*_m_ (°C)
BP	56.8	177.3
BPC	57.1	178.8
BP/GO-0.5	59.8	178.7
BPC/GO-0.5	62.8	180.8
BPC/GO-1.0	65.2	184.2

The results of the phase transition temperature of PA12 in the PA12/PPO blend are completely similar to the arguments and results in the dynamic mechanical analysis. The presence of compatibilizers improved the phase interaction between PA12 and PPO, thereby increasing the glass transition temperature and melting temperature of PA12 (shifting to the phase transition region of PPO). Besides, the interface interaction of PA12 with GO can restrict the polymer chain motion, resulting in enhanced *T*_g_ and *T*_m_ of PA12.

### 3.5. Flammability of PA12/PPO blends

To evaluate the flame resistance of the PA12/PPO blend, the samples were burned vertically according to UL-94 to measure the burning time of the samples. The sample is classified as V0 grade if the burning time *t* is shorter than 10 seconds, and classified as V1, V2 grade if *t* is shorter than 60 seconds with and without dripping, respectively. The Limiting Oxygen Index (LOI) was also determined to assess the minimum oxygen concentration required to sustain combustion.

The incompatible blend (BP) showed a burning time of 58 seconds with drip and achieved a V2 grade. This result indicates a relatively low flame resistance, which is typical of immiscible polymer blends with poor interfacial adhesion. In this sample, the presence of phase separation structures may facilitate localized decomposition, reducing the flame-retardant effect of the PPO phase on the PA12 matrix, leading to dripping during combustion, thereby reducing the flame spread resistance of the material. Moreover, the LOI value of 21.9% further confirms the poor fire resistance of this blend, implying that this material can sustain combustion even under normal air conditions (Table 5).

**Table 5:** Effect of different compatibilizer systems on burning time, grade, and LOI of PA12/PPO blends

Sample	Burning time (seconds)	Grade	LOI
BP	58	V2	21.9
BPC	56	V2	22.1
BP/GO-0.5	51	V1	23.2
BPC/GO-0.5	40	V1	23.7
BPC/GO-1.0	37	V1	24.6

The addition of PPO-*g*-MA slightly reduced the burning time to 56 seconds but did not improve the UL-94 classification, which remained at V2 grade. This suggests that while PPO-*g*-MA enhances interfacial adhesion and overall mechanical properties, it does not significantly alter the combustion pathway or contribute to char formation. Therefore, its effect on flame retardancy is limited when used alone. However, the slower thermal decomposition (confirmed by TGA results) and better compatibility (confirmed by DMA and DSC results) resulted in a modest improvement in the material's fire resistance, as shown by the increased LOI value (>22% – surpassing the stage of flammable materials).

In contrast, the incorporation of 0.5 wt% graphene oxide in the BP/GO-0.5 sample led to a significant improvement in flame-retardant performance. The burning time decreased to 51 seconds without a drip and an LOI value of 23.2%. This enhancement is attributed to the intrinsic flame-retardant characteristics of GO. During combustion, GO not only reduces phase separation in the blend but also combines with the ash layer generated by PPO, acting as a thermal barrier that slows heat release and gas diffusion, while simultaneously promoting the formation of a protective char layer that insulates the underlying material. In addition, the high thermal conductivity and stability of GO can provide more effective heat dissipation, reducing the rate of decomposition.

This effect is even more pronounced with blends containing both compatibilizers. The BPC/GO-0.5 and BPC/GO-1.0 samples showed significantly lower burning times of 40 and 37 seconds, respectively, without drip. The synergistic interaction between PPO-*g*-MA and GO contributes to this improvement. PPO-*g*-MA enhances phase compatibility, leading to more uniform morphology and consistent char formation, while GO serves as an effective heat shield and gas barrier during combustion. Notably, the BPC/GO-1.0 sample exhibited the highest LOI value of 24.6%, indicating a remarkable improvement in flame resistance compared to the other blends. This result clearly correlates with the high residual char yield (5.3%) obtained from the TGA analysis. The greater char formation suggests that during combustion, the presence of a higher GO content facilitates the development of a dense and thermally stable carbonaceous layer on the material surface. The reduction in dripping, observed during the tests, is likely due to the cohesive structure formed by the compatibilized matrix and the presence of GO-reinforced char residue.

These results confirm that the combination of PPO-*g*-MA and GO not only improves mechanical and thermal properties but also contributes significantly to the flame retardancy of the PA12/PPO blends. However, the flame-retardant performance of the material is still limited and needs to be improved by adding some other flame-retardant additives to meet the flame-retardant requirements of practical products.

## 4 Conclusions

PA12/PPO blend (90/10 wt%) was prepared, and the compatibility between the two phases was enhanced by using PPO-*g*-MA, graphene oxide, and their combinations, to enhance the interfacial compatibility and improve the overall performance of the blend. Morphological investigation showed that the combined use of PPO-*g*-MA and GO produced a synergistic effect, improving the interaction performance between the phases in the blend, resulting in significantly better overall performance than using each compatibilizer individually. The shift of *T*_g_ in the blends containing the dual compatibilizer system further confirmed their compatibility role. As a result, the mechanical properties, thermal stability, and flame retardancy of the PA12/PPO blend were significantly improved when using the combination of PPO-*g*-MA and GO. The BPC/GO-1.0 sample exhibited a tensile strength of 48.6 MPa and an impact strength of 47.3 kJ m^−2^, respectively. The material has a higher modulus (1.69 GPa) while maintaining good flexibility, showing a good balance between stiffness and toughness. It had improved thermal stability (*T*_10%_ = 385.8 °C, 53.4 °C higher than the neat blend) with a superior residual char rate (5.3% compared with 2.2% of the neat blend), demonstrating the barrier effect of GO and the supportive role of PPO-*g*-MA. Correspondingly, its flame retardancy improved significantly (burning time of 37 seconds, UL-94 V1 rating, LOI of 24.6% *vs.* 58 seconds, UL-94 V2 rating, LOI of 21.9% of the neat blend).

The results of this study confirm the synergistic compatibilization of PPO-*g*-MA and GO in the preparation of high-performance PA12/PPO blends. These findings open up a promising strategy for the development of new materials that can meet the higher requirements of the manufacturing industry.

## Author contributions

Conceptualization, Luong Nhu Hai, Pham The Long; data analysis, Pham The Long, Nguyen Thi Ngoan, Vu Thi Hoang Anh, Nguyen Huu Dat; methodology, Pham The Long, Luong Nhu Hai, Nguyen Thi Ngoan; project administration, Nguyen Thi Ngoan; validation, Luong Nhu Hai, Nguyen Vu Giang, Nguyen Thi Ngoan, and Pham The Long; writing – original draft, Nguyen Thi Ngoan, Pham The Long; writing – review & editing, Nguyen Thi Ngoan, Pham The Long, Tran Thi Y Nhi and Luong Nhu Hai. All authors have read and agreed to the published version of the manuscript.

## Conflicts of interest

The authors declare that they have no conflict of interest.

## Data Availability

All data generated or analyzed in this study are included in the article. Additional datasets are available from the corresponding author upon reasonable request. There are no restrictions on data sharing, and all data can be made available to the scientific community for future research.
